# Isolation, Characterization, and Stability Assessment of Pure Enantiomers of Cathinone Derivatives via Semi-Preparative HPLC-UV Using a Phenomenex Lux^®^ 5 Column

**DOI:** 10.3390/molecules31040587

**Published:** 2026-02-08

**Authors:** Stefanie Handl, Katrin Stelzeneder, Annaluna Ravelli, Martin G. Schmid

**Affiliations:** Department of Pharmaceutical Chemistry, Institute of Pharmaceutical Sciences, University of Graz, Schubertstraße 1, 8010 Graz, Austria; stefanie.handl@uni-graz.at (S.H.); katrin.stelzeneder@edu.uni-graz.at (K.S.); 272276@studenti.unimore.it (A.R.)

**Keywords:** synthetic cathinones, enantioseparation, stability studies, semi-preparative HPLC-UV, new psychoactive substances (NPS)

## Abstract

In addition to well-known traditional synthetic illicit drugs like cocaine, amphetamines, and heroin, an increasing number of new psychoactive substances (NPS) are appearing on the global drug market. Among them, cathinones represent a prominent class. These amphetamine-like compounds contain a stereogenic center, resulting in the possible presence of two enantiomers. Pure enantiomers of cathinone derivatives are not commonly available, and their production is cost-intensive. Thus, there is very little knowledge about the possible distinct effects of single enantiomers of cathinones. The objective of this study was to evaluate the stability of a set of eight cathinone derivatives, namely 3-methylethcathinone, 3-methylmethcathinone, 4-methylethcathinone, 4-methylmethcathinone, ethylone, 3,4-trimethylene-α-ethylaminovalerophenone, 3,4-tetramethylene-α-pyrrolidinovalerophenone, and 3,4-trimethylene-α-pyrrolidinobutiophenone, over a six-month period. Any racemization that may have occurred under different storage and solution conditions was monitored and compared. Pure enantiomeric fractions were collected on a multi-milligram scale using semi-preparative HPLC under isocratic normal-phase conditions. A Phenomenex Lux^®^ i-Cellulose-5, 5 μm 250 × 10 mm column containing cellulose tris(3,5-dichlorophenylcarbamate) served as the chiral selector. The tests showed that aqueous conditions, pH, temperature, chemical structure, sunlight, and oxygen influence compound stability. The long-term storage of cathinone derivative enantiomers was found to be optimal as solids under deep-freezing conditions or in a slightly acidified solvent where they are protected from air and light.

## 1. Introduction

New psychoactive substances (NPS) represent a rapidly evolving class of chemical compounds mimicking the desired effects of traditional illicit drugs. They are associated with unpredictable pharmacological profiles, often leading to severe toxicity after consumption [1]. Most of the available information about these compounds comes from progress reports shared by users of these drugs on internet forums. Over the last decade, the rate of synthesis and marketing of NPS has increased dramatically, with more than 1000 new substances being released worldwide through online platforms by 2024. This rapid increase in availability poses a significant challenge to drug control agencies, public health authorities, and scientific researchers. Besides synthetic cannabinoids, synthetic stimulants, new opioids, and other substances classified by the European Union Drugs Agency (EUDA), cathinones are one of the main NPS categories [2,3,4,5]. They represent derivatives of S-cathinone, the parent compound present in the khat plant (*Catha edulis*), which is an evergreen shrub native to parts of Africa and the Arabian Peninsula [6]. The cultivation and trade of khat leaves are highly profitable, resulting in widespread terraced plantations in countries such as Yemen and Ethiopia [7]. Besides cathinone, khat contains the less active compound cathine (norpseudoephedrine). In general, cathinones exhibit stimulant effects similar to amphetamines [8]. During the 2000s, various cathinones started to be traded worldwide as “legal highs”. To avoid legal restrictions, these substances are often labeled as “not for human consumption” or “for research purposes only”. New derivatives are constantly being developed to fulfill the demand for legal alternatives to traditional drugs [9,10]. The presence of a stereogenic center in cathinone derivatives results in the existence of two enantiomeric forms, each potentially exhibiting distinct pharmacological and toxicological effects. For this reason, there is a high demand for scientific studies that investigate structure–activity relationships and the development of special chiral analytical methods for the separation and isolation of NPS enantiomers [11].

To date, besides mephedrone, methcathinone, and MDPV, no cathinone derivative enantiomer is commercially available, neither as a standard compound nor from an internet shop. With the use of chiral separation methods of cathinones, e.g., by means of HPLC, commercially available racemates of cathinones can be resolved into their enantiomers. They can be collected to be the subject of enantiospecific tests. However, one must be aware that these enantiomers tend to undergo racemization, which must be controlled. The findings of these studies could significantly influence future research in the field of NPS analysis [12]. In the literature, studies have confirmed the different potencies of individual enantiomers of illicit drugs, including methamphetamine [13], methcathinone [14,15,16], mephedrone [16,17,18], ethylone [19,20], and amphetamine [21]. In the publications detailed below, considerable progress in the enantiomeric separation of NPS on an analytical scale has been made. With the use of techniques such as high-performance liquid chromatography (HPLC) [1,22,23], capillary electrochromatography (CEC) [24], capillary electrophoresis (CE) [25,26,27,28], gas chromatography (GC) [29], and supercritical fluid chromatography (SFC) [24,30], reliable methods for the identification of NPS have been reported both for solid samples and biological matrices. Only a few studies have addressed the isolation of pure NPS enantiomers. In 2016, Silva et al. isolated the enantiomers of methylenedioxypyrovalerone (MDPV) successfully, a cathinone derivative classified as an NPS that acts as a stimulant by inhibiting the reuptake of norepinephrine and dopamine. To achieve the enantioseparation of MDPV, the authors employed semi-preparative HPLC with UV detection, using an amylose tris(3,5-dimethylphenylcarbamate) column as the chiral selector [31]. In 2018, the enantiomers of the cathinone derivatives pentedrone and methylone were isolated by the same research group using an amylose tris [(S)-α-methylbenzylcarbamate] column [32]. More recently, in 2023, Almeida et al. reported the enantioseparation of fourteen synthetic cathinones. The collected pure enantiomers were used to complete binding studies of these cathinones to human serum albumin using high-performance affinity chromatography [33]. Chiral NPS are typically found as a racemic mixture, since stereoselective synthesis is both challenging and expensive. Underground laboratories, particularly those located in Eastern Europe and Asia, have developed diverse methods to produce a wide range of unknown compounds. Consequently, more harmful high-purity NPS enantiomers may be produced and sold in the future [3,34]. To control the purity of enantiomers, the term enantiomeric excess (ee) is widely used as a reference value. This fundamental concept in stereochemistry quantifies the purity of chiral compounds by measuring the relative amount of one enantiomer over the other in a mixture, and it emphasizes the degree of predominance of each enantiomer [35]. In contrast, the enantiomeric ratio (e.r.) expresses the relative amounts of the two enantiomers, providing a direct comparison of both forms [36]. Enantiomeric stability tests are also crucial for cathinones because the enantiomeric ratio (e.r.) can change during storage, potentially leading to inaccurate purity results [37]. This finding is significant for chiral psychoactive substances, since the potentially dangerous effects of the two enantiomers can differ.

The goal of this study was to isolate pure cathinone enantiomers of eight selected racemates traded via the internet, namely 3-methylethcathinone (3-MEC), 3-methylmethcathinone (3-MMC), 4-methylethcathinone (4-MEC), 4-methylmethcathinone (4-MMC, mephedrone), ethylone, 3,4-trimethylene-α-ethylaminovalerophenone (bk-iVP), 3,4-tetramethylene-α-pyrrolidinovalerophenone (TH-PVP), and 3,4-trimethylene-α-pyrrolidinobutiophenone (5-PPDI), using a semi-preparative chiral HPLC-UV method and following a protocol previously used for the analytical-scale enantioseparation of various NPS [2]. To answer the question of the stability of these collected enantiomers, they were stored at three different common storage temperatures for a period of six months. The results of the compounds, dissolved in water, methanol, and as a neat substance, were compared. To control for potential racemization, a polarimeter was used to measure the optical rotation of each fraction.

## 2. Results

In recent years, polysaccharide-based columns have been extensively evaluated for the optimization of chiral separations of NPS. Robust analytical methods have been used to scale up the use of a semi-preparative method to access isolated enantiomers of NPS. Initial experiments with a Phenomenex Lux^®^ 2 column (5 μm, 250 × 10 mm) under polar conditions used by Hägele et al. (unpublished results) did not afford satisfactory resolution because of the difficulty of evaporating aqueous solvents, which might lead to decomposition of the products.

### 2.1. Separation Experiments

**Figure 1:**
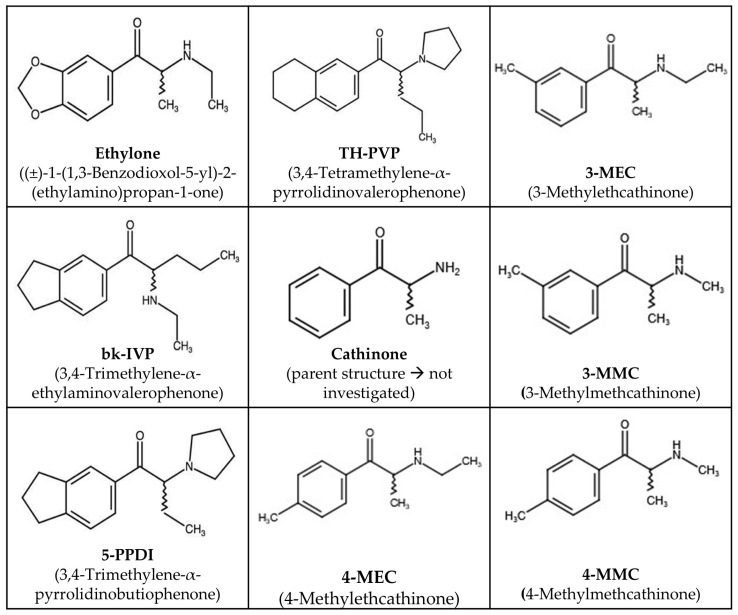
Chemical structures of the investigated NPS with cathinone as the parent structure in the middle of the figure.

A method reported by Kadkhodaei et al. [2] for successful analytical-scale chiral separations of NPS using a Phenomenex Lux^®^ Cellulose 5 column was chosen for a semi-preparative scale-up of the method for pure enantiomer isolation. The mobile phase consisted of n-hexane/IPA/DEA (95:5:0.1), and this was used in isocratic semi-preparative experiments without further modification. Figure 1 presents the chemical structures of all of the investigated chiral cathinone derivative samples, with the parent structure, cathinone, which was not investigated, shown in the center. They were chosen because of their high separation factors and chromatographic resolution. In Table 1, the elution times, separation factors, and chromatographic resolutions of the investigated cathinone derivatives are outlined. The separation times were found to be less than 15 min under the selected conditions.

### 2.2. Purity Control of Enantiomers and Determination of the Specific Optical Rotation

**Figure 2:**
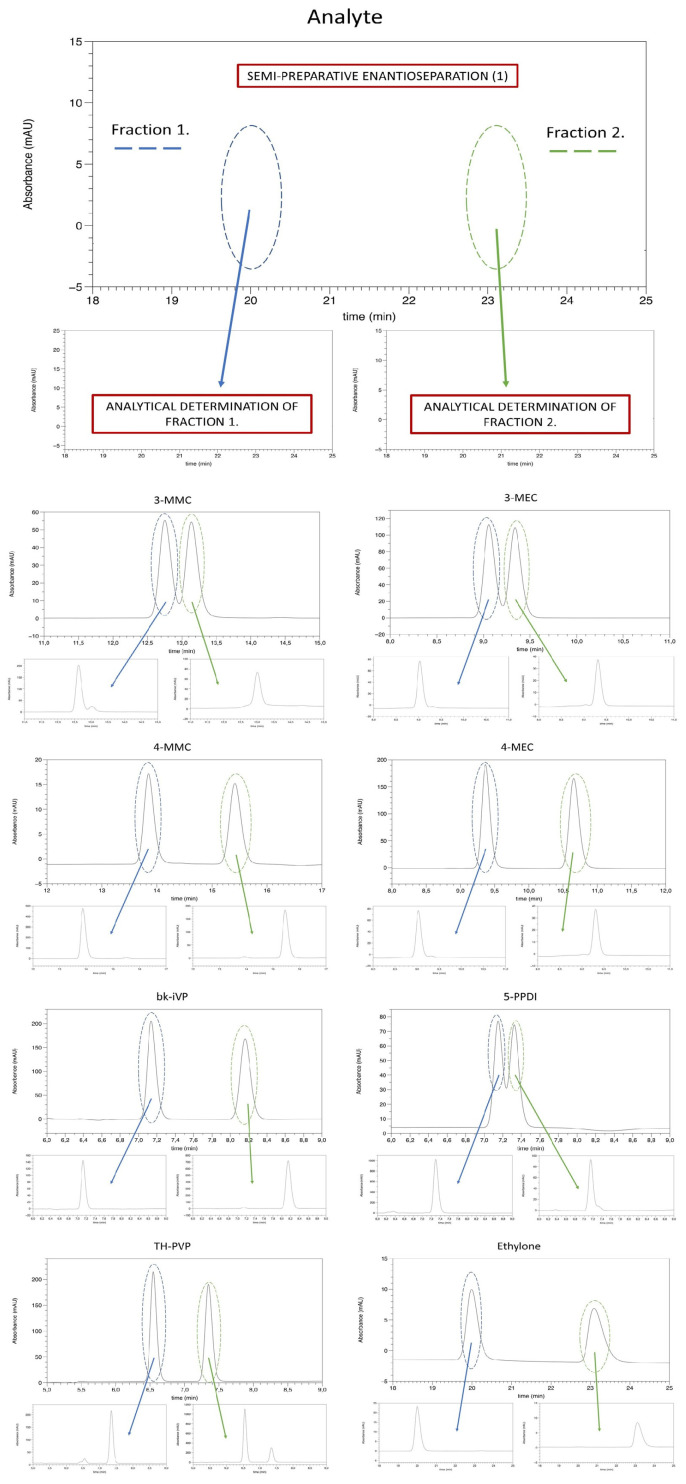
Chromatograms of semi-preparative isolation [2]. Column: Phenomenex Lux^®^ i-Cellulose-5, 5 μm 250 × 10 mm, mobile phase: n-hexane/isopropanol/DEA (100%) (95:5:0.1), ambient temperature, injection: 500 μL, UV: 254 nm and analytical-scale determination of the enantiomeric ratio; legend and detailed description at the top.

The specific optical rotation of each free base of the collected enantiomer was determined using a Jasco P-2000 polarimeter (Ishikawa-machi, Hachioji, Tokyo 192-8537, Japan) in an n-hexane solution at 20 °C. No defined concentrations were applied, as the measurements were only used to determine the optical rotation. The optical rotation was determined solely as a qualitative method of support. It was used to provide complementary information on the chiral nature of the compounds, but it was not considered sufficient for the quantitative assessment. The enantiomeric purity was determined exclusively using the established and validated HPLC method, which ensures accurate, reliable, and reproducible quantification of enantiomeric composition. Corresponding results are summarized in Table 2. Following the method described by Seibert et al. [38] and Kadkhodaei et al. [2], peak areas were determined, and the enantiomeric ratios (e.r.%) were calculated to assess enantiomeric purity. The corresponding results are also presented in Table 2. All enantiomers, except for two cases, showed a purity greater than 90%. Representative chromatograms are provided in Figure 2.

### 2.3. Stability Experiments

**Figure 3:**
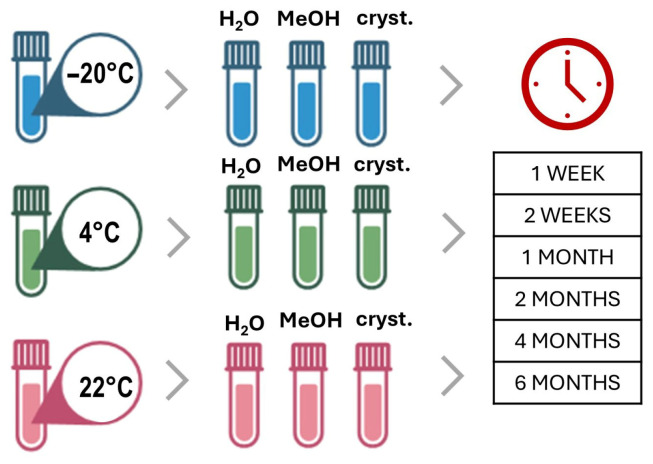
Storage conditions for stability experiments.

Nine brown glass vials were filled with 1 mg of each NPS enantiomer sample to test their stability under various conditions. There was a total of three samples: one was dissolved in 1 mL of deionized water, one was dissolved in 1 mL of methanol, and one contained the solid substance only. The samples were then stored for six months at the following temperatures: ambient temperatures of 22 °C, 4 °C, and −20 °C were used for the sample containing solid material, the one with the aqueous solution, and the one with the methanolic solution, respectively. On the day of isolation, as well as after one and two weeks, and then after one, two, four, and six months, the samples were analyzed using the analytical HPLC method, as shown in Figure 3. For this purpose, the solid samples were dissolved in 1 mL of isopropanol using an ultrasonic bath. After the measurement was performed, isopropanol was evaporated under a stream of nitrogen to recover the solid material. The intra- and interday repeatability data for the model substance 4-MMC with respect to the elution times of the enantiomers and chromatographic resolution are outlined in Table S1.

**Figure 4:**
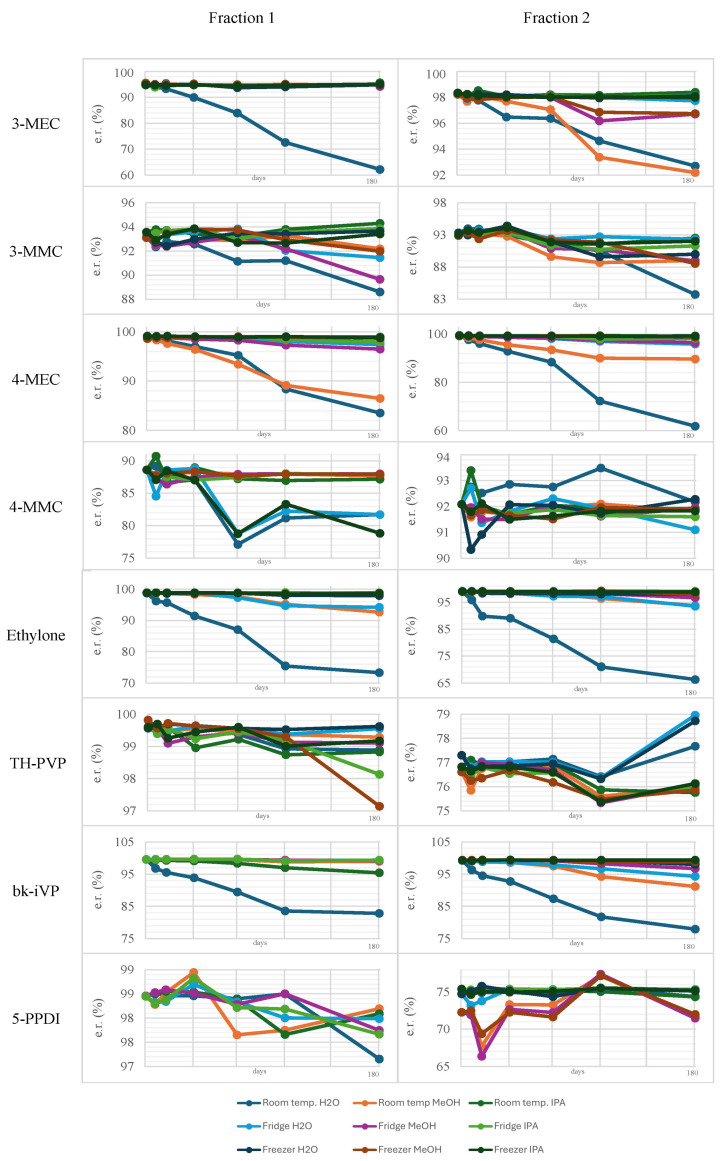
Change in the enantiomeric ratio (%) over a time period of six months from all investigated cathinone derivatives using NPS using a Phenomenex Lux i-Cellulose-5 in an analytical-scale column. Conditions: Column: Phenomenex Lux^®^ i-Cellulose-5, 3,5 μm 250 × 10 mm, mobile phase: n-hexane/isopropanol/DEA (100%) (95:5: 0.1), ambient temperature, injection: 1 μL, UV: 254 nm.

Regarding the stability of the tested cathinone derivative enantiomers, use of the presented preparative HPLC method has proven to be successful for most of the chosen NPS. However, for TH-PVP and 5-PPDI, further optimization is still required to separate both enantiomeric fractions with a high degree of purity. This might inevitably lead to a significant reduction in yield. In the subsequent stability tests, only the room-temperature samples were examined for their initial value. Slightly elevated or fluctuating values may also indicate minor errors in the evaluation, as many of the determined chromatogram peaks required manual readout due to stronger baseline noise observed in the methanol samples. The results over a six-month period are shown in Figure 4.

## 3. Discussion

**Figure 5:**
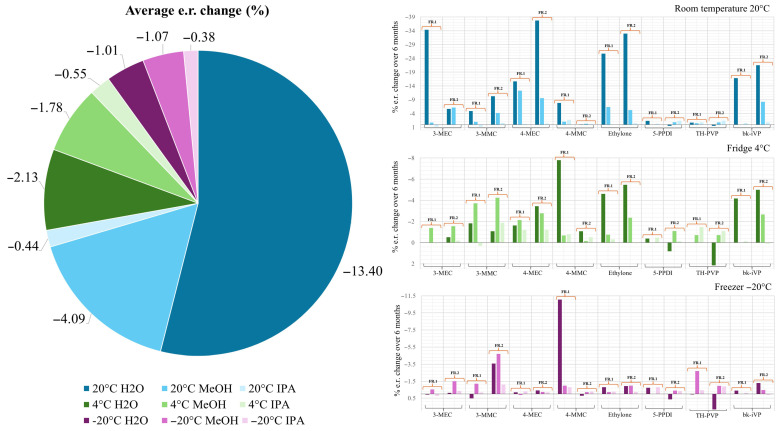
Overview of the stability tests with a pie chart (**left**) illustrating the overall distribution and bar charts (**right**) showing the results for each temperature condition.

It is hypothesized that cathinones differ in their tendency to re-racemize due to the presence of a carbonyl group, which can exist in keto or enol form (keto-enol tautomerism). Acidic conditions may favor the keto form through protonation of oxygen, while basic conditions could promote enol formation via deprotonation of α-carbon. In general, the keto form is thermodynamically more stable, but its stability can be further enhanced by adjacent aromatic systems or intra- and intermolecular hydrogen bonding. As a general rule, the more highly substituted the enol molecule is, the more stable it becomes [39]. Both water and methanol represent neutral to slightly basic solvents. The predominance of the enol form under these conditions may contribute to the reduced stability of the cathinone derivatives dissolved in these solvents. The incorporation of an acid may significantly enhance this process, as clearly demonstrated by the findings of Yang et al. [40]. In this study, various synthetic cathinones were dissolved in a mixture of equal parts of water and ethanol. While the addition of 0.1% formic acid to the samples increased stability, a significant decrease in stability was observed in the samples that were dissolved in either water or ethanol only. Our findings confirmed that the samples dissolved in water showed instabilities in the case of 4-MEC, ethylone, and bk-iVP. A high water content typically promotes degradation via hydrolysis. Using the established HPLC method, the formation of degradation products was progressively more apparent, allowing for their monitoring over the course of the study. The functional groups of cathinones, such as the carbonyl and amino residues, are not sensitive to hydrolysis at a neutral pH. Moreover, temperature also plays a significant role in molecular stability. Consequently, the reaction rate rises exponentially with the increase in temperature. This phenomenon was also observed in this work, as shown in Figure 5. While the samples at −20 °C showed, in most cases, similar enantiomeric ratios to the starting values even after six months of storage, such as 4-MEC and bk-iVP, they underwent almost complete racemization within the same time period. However, racemization depends not only on temperature but also on the type of cathinone derivative and the chosen solvent. Different temperatures significantly affect the results of stability studies; this has been confirmed by Adamowicz and Malczyk [41]. In their research, the authors stored various synthetic cathinones in blood and urine at three different temperatures, namely 24 °C, 5 °C, and −26 °C. By measuring the decomposition grade of the tested substances, they determined the half-life of different dissolved cathinone derivatives. The half-life of the samples stored at room temperature varied from a few days to a few months. In contrast, very few of the samples stored at −20 °C had degraded by half after a six-month testing period. Some of the deep-frozen cathinone derivatives showed half-lives of over three years. Notably, a further stability factor is the chemical structure of each cathinone derivative.

All substance samples exhibited different stabilities when exposed to the same conditions. While 4-MEC, 3-MEC, and bk-IVP showed, on average, significant decreases in enantiomeric ratio, the cathinones 3-MMC, TH-PVP, and 5-PPDI were found to be more stable. One example of a structural difference that can impact stability is the type of amine substitution. According to a publication by Tsujikawa et al., secondary amines are highly susceptible to oxidative deamination, whereas this is rarely seen with tertiary-substituted amines [42]. This may explain why the cathinone derivatives with a pyrrolidine ring, namely TH-PVP and 5-PPDi, exhibited comparatively higher stability during the two-month storage period. In the same study by Tsujikawa et al., it was found that the cathinone derivative 4-MMC remains stable for a longer period when antioxidants are added compared with the pure substance solution in airtight sample vials. This suggests that even small amounts of dissolved atmospheric oxygen in the sample solution may have a significant impact on the stability of cathinone derivatives. Furthermore, according to Ciallella et al., condensed benzenes also influence stability [43]. This study showed that the presence of this structural element leads to increased electrophilicity in the molecule and thus to reduced stability. However, this could not be clearly confirmed in the stability tests conducted in this study since none of the substances tested possess a condensed benzene in their structure. In Figure 5, an overview of all the stability tests is presented. The samples stored at room temperature, particularly those dissolved in water, exhibit the most pronounced decrease in their enantiomeric ratio. In contrast, the cryogenically stored samples show only minimal changes. Furthermore, for TH-PVP and 5-PPDI, both tertiary amines, virtually no alteration is observed.

In summary, enantiomers of cathinone derivatives should be stored in a deep-frozen manner as solid substances or in a slightly acidified solvent, protected from air and light, to ensure that the best possible stability is achieved. However, optimal storage conditions are no guarantee of high stability since the chemical composition of the cathinone derivative itself also plays a significant role in stability.

## 4. Materials and Methods

### 4.1. Chemicals

All chemicals used were of analytical grade. Methanol (MeOH) and isopropanol (IPA) were obtained from VWR Chemicals (Darmstadt, Germany). Diethylamine (DEA) was purchased from Sigma-Aldrich (St. Louis, MO, USA), and n-hexane was obtained from VWR International (Fontenay-sous-Bois, France). Aqueous sodium hydroxide solution (2 M) and concentrated sulfuric acid were supplied in-house. Due to the limited availability of most analytes from official chemical suppliers, they were bought from various online sources or were real-life samples seized by Austrian police. Before their use in chiral separation experiments, the identity of all analytes was verified using gas chromatography–mass spectrometry (GC-MS) (Agilent 5301 Stevens Creek Blvd. Santa Clara, CA 95051, USA), infrared spectroscopy (IR): (Anton-Paar, Anton-Paar-Straße 20, 8054 Graz, Austria), and, when necessary, nuclear magnetic resonance spectroscopy (NMR)(Bruker Avance Neo NMR, Bruker, Rheinstetten, Germany). All analytes were present as hydrochloric salts.

### 4.2. Sample Preparation for Semi-Preparative Experiments

Each racemic cathinone sample was prepared individually at concentrations ranging from 5 mg/mL to 20.0 mg/mL. The hydrochloric salt of the sample was dissolved in 1 mL of deionized water and subsequently treated with 0.1 mL of an aqueous sodium hydroxide solution (2 M). Then, 10 mL of n-hexane was added and thoroughly shaken. After this liquid–liquid extraction, the upper n-hexane phase was removed, yielding the free base of the cathinone racemates. For conversion of the free bases to their hydrochloric salt, the mobile phase of all collected enantiomer fractions was evaporated under reduced pressure at 40 °C. The resulting free base was redissolved in n-hexane and treated with dry hydrochloric acid gas, resulting in the precipitation of each enantiomer as its hydrochloride salt. Finally, the residual n-hexane was again removed via evaporation.

### 4.3. Instrumentation and Chromatographic Conditions for Collection of Pure Enantiomers

Semi-preparative separations were conducted on an Äkta Explorer Liquid Chromatograph (Amersham Pharmacia Biotech, Uppsala, Sweden) equipped with a 500 μL loop injector, an individual fraction collection system, and a diode array detector. UV absorption was monitored at a wavelength of 254 nm. After each run, the enantiomer fractions were collected and pooled. In total, 50–70 runs were required to obtain a representative amount of pure enantiomers. As a chiral stationary phase, a Phenomenex Lux^®^ i-Cellulose-5, 5 μm 250 × 10 mm column (Aschaffenburg, Germany) was used. The mobile phase for the semi-preparative isolation consisted of n-hexane/IPA/DEA (95:5:0.1). After preparing the mobile phase, a degassing step was carried out in an ultrasonic bath for at least 2 min. The collection of pure enantiomers was performed under isocratic conditions and at room temperature (25 ± 1 °C). Flow rates of 4 mL/min to 7 mL/min were applied during the measurements, depending on the substance. For data acquisition, the software Unicorn 5.31 was used. The optical rotation of each enantiomer fraction was determined using a Jasco P-2000 polarimeter at 20 °C in n-hexane.

### 4.4. Determination of Enantiomeric Ratio Using Analytical HPLC

For the determination of the enantiomeric ratio, all measurements were performed using an analytical Agilent 1260 Infinity II liquid chromatograph (5301 Stevens Creek Blvd, Santa Clara, CA 95051, United States), equipped with a variable-wavelength detector and an autosampler. Analyses were performed under isocratic conditions at room temperature (25 ± 1 °C), an injection volume of 1 μL, and a flow rate of 1.0 mL/min. UV absorption was recorded at a wavelength of 254 nm. For data acquisition, the software Open-Edition Rev. C. 01.07SR2 [255] for LC and LC/MS Systems, by Agilent Technologies (Waldbronn, Germany), was used. A Phenomenex Lux^®^ i-Amylose-3, 3 μm, 250 × 4.6 mm column and a Phenomenex Lux^®^ i-Cellulose-5, 3.5 μm, 250 × 4.6 mm column (Aschaffenburg, Germany) were used as chiral selectors [1,38]. The areas of both peaks were subject to the enantiomeric ratio (e.r.). The e.r. was calculated from the relative peak areas, expressed as percentages, where [E1] and [E2] correspond to the peak areas of each enantiomer [36]:

$$e.r.\left(\%\right)=100\;\frac{\left[E1\right]}{\left[E1\right]+\left[E2\right]}\;or\;100\;\frac{\left[E2\right]}{\left[E1\right]+\left[E2\right]}$$

## 5. Conclusions

Many NPS misused as illicit drugs are chiral, which may result in their enantiomers having distinct toxicological and pharmacological effects. Currently, there is only a minimal amount of knowledge regarding the properties of individual NPS enantiomers, although existing data on a few representative compounds suggest that such differences are likely. Since pure NPS enantiomers are expensive or unavailable from commercial suppliers, their synthesis or isolation is essential for the performance of further pharmacological and toxicological studies. Using this method, the enantiomers of eight racemic NPS available on the drug market were isolated using a semi-preparative HPLC-UV and a Lux^®^ 5, 5 μm, 250 × 10 mm column. The mobile phase of n-hexane/IPA/DEA (100%) (95:5:0.1) proved to be optimal, even in the context of connected solvent removal. The use of the presented semi-preparative HPLC method has proven to be reliable for this purpose. However, if resolution is not satisfactory, further optimization is required to achieve high-purity separation of both fractions. This adjustment, though, inevitably results in a considerable decrease in yield. To our knowledge, the NPS enantiomers of bk-iVP, TH-PVP, 5-PPDI, and ethylone were isolated for the first time. Additionally, the specific optical rotations of the enantiomers were identified. The isolated enantiomers were applied to stability tests using analytical HPLC-UV with different separation modes. Stability studies have demonstrated that a variety of external and intrinsic factors strongly influence the preservation of cathinone derivatives. Among the most critical are the presence of water, the pH of the solution, temperature, the specific chemical structure of the individual cathinone derivative, and environmental influences such as exposure to sunlight and atmospheric oxygen. Each of these parameters can accelerate degradation processes, thereby compromising both the chemical integrity and the enantiomeric purity of the compounds. For this reason, the long-term storage of cathinone derivative enantiomers is most effectively achieved in neat form under deep-freezing conditions, where racemization reactions are significantly slowed down. Alternatively, storage in a slightly acidified solvent under an inert and light-protected environment can also provide a suitable means of maintaining stability over extended periods of time.

## Figures and Tables

**Table 1:** Chiral separation data of the investigated NPS using Phenomenex Lux i-Cellulose-5 in a semi-preparative scale column. Conditions: Column: Phenomenex Lux^®^ i-Cellulose-5, 5 μm 250 × 10 mm, mobile phase: n-hexane/isopropanol/DEA (100%) (95:5:0.1), ambient temperature, injection: 500 μL, UV: 254 nm.

Compound	t1 (min)	t2 (min)	α	Rs	Flowrate (mL/min)	Amount/ Injection (mg in Salt Form)
3-MEC	5.66	6.54	1.44	5.94	7.0	10.0
3-MMC	8.53	9.33	1.16	6.26	7.0	10.0
4-MEC	6.56	7.88	1.33	5.84	7.0	10.0
4-MMC	11.20	12.55	1.19	4.86	7.0	7.5
5-PPDI	7.01	7.51	1.27	9.07	5.0	10.0
bk-iVP	4.35	5.16	1.77	10.56	7.0	2.5
TH-PVP	6.49	7.41	1.83	6.89	7.0	10.0
Ethylone	11.00	12.91	1.27	14.98	7.0	7.5

**Table 2:** Optical rotation and enantiomeric ratio after the isolation of the investigated cathinone derivatives as their free bases.

Analyte	Enantiomer	Optical Rotation	% e.r.
3-MMC	E1	−	93.56
	E2	+	93.06
3-MEC	E1	−	94.87
	E2	+	98.33
4-MMC	E1	+	88.63
	E2	−	92.08
4-MEC	E1	−	99.12
	E2	+	99.47
Ethylone	E1	−	98.89
	E2	+	99.04
TH-PVP	E1	−	99.10
	E2	+	76.82
5-PPDI	E1	−	98.45
	E2	+	75.39
bk-iVP	E1	+	99.49
	E2	−	99.25

## Data Availability

Data is contained within the article or supplementary material.

## Supplementary Materials

The following supporting information can be downloaded at: https://www.mdpi.com/article/10.3390/molecules31040587/s1. Table S1: Intra- and interday repeatability measurements for the model substance 4-MMC.

## References

[1] Araújo A.M., Valente M.J., Carvalho M., Da Dias Silva D., Gaspar H., Carvalho F., Lourdes Bastos M.d., Guedes de Pinho P. (2015). Raising awareness of new psychoactive substances: Chemical analysis and in vitro toxicity screening of ‘legal high’ packages containing synthetic cathinones. Arch. Toxicol..

[2] Kadkhodaei K., Forcher L., Schmid M.G. (2018). Separation of enantiomers of new psychoactive substances by high-performance liquid chromatography. J. Sep. Sci..

[3] EUDA European Drug Report Trends and Developments, New Psychoactive Substances 2024. https://www.euda.europa.eu/publications/european-drug-report/2025/new-psychoactive-substances_en.

[4] Adley M., Jones G., Measham F. (2023). Jump-starting the conversation about harm reduction: Making sense of drug effects: Thedrugswheel. Drugs Educ. Prev. Policy.

[5] Adley M., Jones G., Linnell M. The Drugs Wheel: A New Model for Substance Awareness. https://www.thedrugswheel.com/.

[6] Al-Hebshi N.N., Skaug N. (2005). Khat (*Catha edulis*)—An updated review. Addict. Biol..

[7] Angelika P. (2009). Bewusstseinsverändernde Pflanzen von A–Z.

[8] Valente M.J., Guedes de Pinho P., Lourdes Bastos M.d., Carvalho F., Carvalho M. (2014). Khat and synthetic cathinones: A review. Arch. Toxicol..

[9] Soares J., Costa V.M., Bastos M.d.L., Carvalho F., Capela J.P. (2021). An updated review on synthetic cathinones. Arch. Toxicol..

[10] Kuropka P., Zawadzki M., Szpot P. (2023). A review of synthetic cathinones emerging in recent years (2019–2022). Forensic Toxicol..

[11] Almeida A.S., Silva B., Pinho P.G.d., Remião F., Fernandes C. (2022). Synthetic Cathinones: Recent Developments, Enantioselectivity Studies and Enantioseparation Methods. Molecules.

[12] Hellwich K.-H. (2007). Stereochemie: Grundbegriffe.

[13] Jirovský D., Lemr K., Sevcík J., Smysl B., Stránský Z. (1998). Methamphetamine—properties and analytical methods of enantiomer determination. Forensic Sci. Int..

[14] Glennon R.A., Dukat M. (2017). Structure-Activity Relationships of Synthetic Cathinones. Neuropharmacology of New Psychoactive Substances (NPS).

[15] Glennon R.A., Young R., Martin B.R., Dal Cason T.A. (1995). Methcathione (“cat”): An enantiomeric potency comparison. Pharmacol. Biochem. Behav..

[16] Schifano F., Albanese A., Fergus S., Stair J.L., Deluca P., Corazza O., Davey Z., Corkery J., Siemann H., Scherbaum N. (2011). Mephedrone (4-methylmethcathinone; ‘meow meow’): Chemical, pharmacological and clinical issues. Psychopharmacology.

[17] Gregg R.A., Baumann M.H., Partilla J.S., Bonano J.S., Vouga A., Tallarida C.S., Velvadapu V., Smith G.R., Peet M.M., Reitz A.B. (2015). Stereochemistry of mephedrone neuropharmacology: Enantiomer-specific behavioural and neurochemical effects in rats. Br. J. Pharmacol..

[18] Saha K., Partilla J.S., Lehner K.R., Seddik A., Stockner T., Holy M., Sandtner W., Ecker G.F., Sitte H.H., Baumann M.H. (2015). ‘Second-generation’ mephedrone analogs, 4-MEC and 4-MePPP, differentially affect monoamine transporter function. Neuropsychopharmacol. Off. Publ. Am. Coll. Neuropsychopharmacol..

[19] Maheux C.R., Alarcon I.Q., Copeland C.R., Cameron T.S., Linden A., Grossert J.S. (2016). Identification of polymorphism in ethylone hydrochloride: Synthesis and characterization. Drug Test. Anal..

[20] Lin H.-R., Kuo F.-W. (2020). Determination of the R- and S-enantiomers of methylone and ethylone in seized drugs by enantioselective liquid chromatography tandem mass spectrometry analysis. Forensic Sci. Int..

[21] Rasmussen L.B., Olsen K.H., Johansen S.S. (2006). Chiral separation and quantification of R/S-amphetamine, R/S-methamphetamine, R/S-MDA, R/S-MDMA, and R/S-MDEA in whole blood by GC-EI-MS. J. Chromatogr. B Anal. Technol. Biomed. Life Sci..

[22] Kadkhodaei K., Kadisch M., Schmid M.G. (2020). Successful use of a novel lux^®^ i-Amylose-1 chiral column for enantioseparation of “legal highs” by HPLC. Chirality.

[23] Schmid M.G., Miolo G., Stair J.L., Zloh M. (2018). Optical Detection of NPS Internet Products via HPLC-DAD Systems: A Selective Review. Light in Forensic Science.

[24] Albals D., Heyden Y.V., Schmid M.G., Chankvetadze B., Mangelings D. (2016). Chiral separations of cathinone and amphetamine-derivatives: Comparative study between capillary electrochromatography, supercritical fluid chromatography and three liquid chromatographic modes. J. Pharm. Biomed. Anal..

[25] Hägele J.S., Hubner E.-M., Schmid M.G. (2019). Chiral separation of cathinone derivatives using β-cyclodextrin-assisted capillary electrophoresis-Comparison of four different β-cyclodextrin derivatives used as chiral selectors. Electrophoresis.

[26] Hägele J.S., Schmid M.G. (2018). Enantiomeric separation of Novel Psychoactive Substances by capillary electrophoresis using (+)-18-crown-6-tetracarboxylic acid as chiral selector. Chirality.

[27] Nowak P.M., Olesek K., Woźniakiewicz M., Kościelniak P. (2018). Simultaneous enantioseparation of methcathinone and two isomeric methylmethcathinones using capillary electrophoresis assisted by 2-hydroxyethyl-β-cyclodextrin. Electrophoresis.

[28] Taschwer M., Hofer M.G., Schmid M.G. (2014). Enantioseparation of benzofurys and other novel psychoactive compounds by CE and sulfobutylether β-cyclodextrin as chiral selector added to the BGE. Electrophoresis.

[29] Weiß J.A., Mohr S., Schmid M.G. (2015). Indirect chiral separation of new recreational drugs by gas chromatography-mass spectrometry using trifluoroacetyl-L-prolyl chloride as chiral derivatization reagent. Chirality.

[30] Pauk V., Žihlová V., Borovcová L., Havlíček V., Schug K., Lemr K. (2015). Fast separation of selected cathinones and phenylethylamines by supercritical fluid chromatography. J. Chromatogr. A.

[31] Silva B., Fernandes C., Tiritan M.E., Pinto M.M.M., Valente M.J., Carvalho M., Pinho P.G.d., Remião F. (2016). Chiral enantioresolution of cathinone derivatives present in “legal highs”, and enantioselectivity evaluation on cytotoxicity of 3,4-methylenedioxypyrovalerone (MDPV). Forensic Toxicol..

[32] Silva B., Pereira J.A., Cravo S., Araújo A.M., Fernandes C., Pinto M.M.M., Pinho P.G.d., Remião F. (2018). Multi-milligram resolution and determination of absolute configuration of pentedrone and methylone enantiomers. J. Chromatogr. B Anal. Technol. Biomed. Life Sci..

[33] Sofia Almeida A., Cardoso T., Cravo S., Elizabeth Tiritan M., Remião F., Fernandes C. (2023). Binding studies of synthetic cathinones to human serum albumin by high-performance affinity chromatography. J. Chromatogr. B Anal. Technol. Biomed. Life Sci..

[34] Hancu G., Modroiu A. (2022). Chiral Switch: Between Therapeutical Benefit and Marketing Strategy. Pharmaceuticals.

[35] Gawley R.E. (2006). Do the terms “% ee” and “% de” make sense as expressions of stereoisomer composition or stereoselectivity?. J. Org. Chem..

[36] Tiritan M.E., Fernandes C., Maia A.S., Pinto M., Cass Q.B. (2018). Enantiomeric ratios: Why so many notations?. J. Chromatogr. A.

[37] Han J., Wzorek A., Klika K.D., Soloshonok V.A. (2021). Recommended Tests for the Self-Disproportionation of Enantiomers (SDE) to Ensure Accurate Reporting of the Stereochemical Outcome of Enantioselective Reactions. Molecules.

[38] Seibert E., Götz K., Schmid M.G. (2024). Exploring a Lux^®^ i-Amylose-3 column in normal phase and polar-organic mode for chiral separation of cathinone derivatives and pyrovalerones using high-performance liquid chromatography. Chirality.

[39] Sarah Kerrigan P. Improved Detection of Synthetic Cathinones in Forensic Toxicology Samples: Thermal Degradation and Analytical Considerations. https://www.ojp.gov/pdffiles1/nij/grants/249251.pdf.

[40] Yang F.-S., Lee H.-H., Tseng L.-P., Lee Y.-H., Lan Y.-S., Lee Y.-C., Chou Y.-C., Lin Y.-C. (2023). Simultaneous Determination and Stability Analysis of Ten New Psychoactive Substances including Synthetic Cathinones, Phenethylamines, and Ketamine Substitutes in Urine Using Liquid Chromatography-Tandem Mass Spectrometry. Int. J. Anal. Chem..

[41] Adamowicz P., Malczyk A. (2019). Stability of synthetic cathinones in blood and urine. Forensic Sci. Int..

[42] Tsujikawa K., Mikuma T., Kuwayama K., Miyaguchi H., Kanamori T., Iwata Y.T., Inoue H. (2012). Degradation pathways of 4-methylmethcathinone in alkaline solution and stability of methcathinone analogs in various pH solutions. Forensic Sci. Int..

[43] Ciallella H.L., Rutter L.R., Nisbet L.A., Scott K.S. (2020). Extended Stability Evaluation of Selected Cathinones. Front. Chem..

