# Supplementary material for: Isolation, Characterization, and Stability Assessment of Pure Enantiomers of Cathinone Derivatives via Semi-Preparative HPLC-UV Using a Phenomenex Lux® 5 Column
Source: Molecules. 2026 Feb 8;31(4):587. doi: 10.3390/molecules31040587 (PMC12943759; doi:10.3390/molecules31040587)
Supplement: Supplementary file 1 [file molecules-31-00587-s001.zip › molecules-4085666-supplementary.pdf]

Substance		*t_1 (min)*	*t_2 (min)*	*Rs*
4-MMC	Intraday n = 5	11.15 ± 0.09 RSD = 0.63%	12.67 ± 0.10; RSD = 0.67%	4.86 ± 0.13: RSD = 3.52%
	Interday n = 5	11.34 ± 0.17 RSD = 1.25%	12.51 ± 0.15; RSD = 1.04%	4.89 ± 0.07; RSD = 2.07%

Table S1: Intra- and interday repeatability measurements for the model substance 4-MMC.
